# Indole-3-acetic-acid and ACC deaminase producing *Leclercia adecarboxylata* MO1 improves *Solanum lycopersicum* L. growth and salinity stress tolerance by endogenous secondary metabolites regulation

**DOI:** 10.1186/s12866-019-1450-6

**Published:** 2019-04-25

**Authors:** Sang-Mo Kang, Raheem Shahzad, Saqib Bilal, Abdul Latif Khan, Yeon-Gyeong Park, Ko-Eun Lee, Sajjad Asaf, Muhammad Aaqil Khan, In-Jung Lee

**Affiliations:** 1 Institute of Agricultural Science and Technology, Kyungpook National University, Daegu, 41566 Republic of Korea; 2 Basic and Applied Scientific Research Center, Imam Abdulrahman Bin Faisal University, Dammam, Saudi Arabia; 3 School of Applied Biosciences, Kyungpook National University, Daegu, 41566 Republic of Korea; 4 Natural and Medical Sciences Research Center, University of Nizwa, Nizwa, Oman

**Keywords:** PGPR, Tomato, NaCl, Plant microbe interaction, Metabolite regulation

## Abstract

**Background:**

The utilization of plant growth-promoting microbes is an environment friendly strategy to counteract stressful condition and encourage plants tolerance. In this regards, the current study was designed to isolate ACC deaminase and indole-3-acetic acid (IAA) producing halotolerant bacteria to promote tomato (*Solanum lycopersicum* L.) growth and tolerance against salinity stress.

**Results:**

The selected bacterial isolate MO1 was identified as *Leclercia adecarboxylata* and IAA quantification results revealed that MO1 produced significant amount of IAA (9.815 ± 0.6293 μg mL^− 1^). The MO1 showed the presence of ACC (1-Aminocyclopropane-1-Carboxylate) deaminase responsible *acdS* gene and tolerance against salinity stress. A plant microbe interaction experiment using tomato (*Solanum lycopersicum* L.) with glycine betaine (GB) as a positive control was carried out to investigate the positive role MO1 in improving plant growth and stress tolerance. The results indicated that MO1 inoculation and GB application significantly increased growth attributes under normal as well as saline condition (120 mM NaCl). The MO1 inoculation and GB treatment approach conferred good protection against salinity stress by significantly improving glucose by 17.57 and 18.76%, sucrose by 34.2 and 12.49%, fructose by 19.9 and 10.9%, citric acid by 47.48 and 34.57%, malic acid by 52.19 and 28.38%, serine by 43.78 and 69.42%, glycine by 14.48 and 22.76%, methionine by 100 and 124.99%, threonine by 70 and 63.08%, and proline by 36.92 and 48.38%, respectively, while under normal conditions MO1 inoculation and GB treatment also enhanced glucose by 19.83 and 13.19%, sucrose by 23.43 and 15.75%, fructose by 15.79 and 8.18%, citric acid by 43.26 and 33.14%, malic acid by 36.18 and 14.48%, serine by 46.5 and 48.55%, glycine by 19.85 and 29.77%, methionine by 22.22 and 38.89%, threonine by 21.95 and 17.07%, and proline by 29.61 and 34.68% compared to levels in non-treated plants, respectively. In addition, the endogenous abscisic acid (ABA) level was noticeably lower in MO1-inoculated (30.28 and 30.04%) and GB-treated plants (45 and 35.35%) compared to their corresponding control plants under normal condition as well as salinity stress, respectively.

**Conclusion:**

The current findings suggest that the IAA- and ACC-deaminase-producing abilities MO1 can improve plants tolerance to salinity stress.

**Electronic supplementary material:**

The online version of this article (10.1186/s12866-019-1450-6) contains supplementary material, which is available to authorized users.

## Background

The unpredictable climatic changes resulting from the rapidly growing population and anthropogenic activities bring various unwanted environmental stresses that negatively affect agriculture productivity [1]. Environmental changes have worsened the rigorousness of various stressors, which negatively influence the agriculture production at the present Anthropocene Era. Meanwhile, food security is needed to maintain for the growing global population by increasing crop production in sustainable and environment friendly way. Among the various environmental stresses caused by climate change, salinity stress represents a major threat to agriculture productivity [2, 3]. Soil salinity is reported to negatively affect more than 77 million hectares, substantially reducing major crops by more than 50% [4]. Currently, around 20% of cultivated land and 50% of irrigated land is effected by salinity stress [5]. Salinity stress has been shown to negatively affect plant growth agronomically and biochemically by reducing nearly all growth attributes, carbon acclimatization, nitrogen metabolism, and grain yield [6–8]. Salinity has three possible effects on plants, namely interfering with essential nutrient uptake, imparting toxicity because of the higher absorbance of Na^+^ and Cl^−^, and lowering the water potential [9–11]. During osmotic and ionic stress triggered in plants by salt stress, the generation of active oxygen species, including superoxide (O_2_–), hydroxyl radicals (· OH), hydrogen peroxide (H_2_O_2_), and singlet oxygen (1O_2_), is thought to play an important role in inhibiting plant growth; furthermore, the active oxygen species need to be scavenged for the maintenance of normal growth [12, 13].

Salt stress is a serious concern in the areas with optimum climate for tomato cultivation [14]. According to USDA, tomato is moderately sensitive to salt stress [15, 16]. Salinity stress effect tomato growth via morphological, physiological and metabolic changes. However, many researchers reported high level of carbohydrates, amino acids, organic acids and total soluble solids in tomato plants grown in high saline conditions [17, 18].

A variety of methods have been used to improve crop tolerance against salinity stress, including traditional and genetic engineering methods, but one of the most promising methods is the utilization of plant growth-promoting microorganisms to promote stress tolerance and growth [19–21]. Many researchers have confirmed the effectiveness of the utilization of phytobeneficial microorganisms for stress mitigation and plant growth promotion [22–24]. Among plant growth-promoting microorganisms, plant growth-promoting rhizospheric bacteria (PGPR) are of great importance because of their direct association with plant roots [25]. PGPR are able to colonize roots, facilitate growth, and mitigate various stresses either directly by producing phytohormones (Gibberellins (GAs), Abscisic acid (ABA), Indole-3-acetic-acid (IAA)), improving nutrient uptake, producing siderophores, and solubilizing minerals or indirectly by decreasing plant pathogens [25, 26]. Various PGPR have been found to mitigate salt stress, improve growth, and enhance tolerance in various crop plants [27–30].

ACC deaminase and IAA producing bacteria assist plant growth and can effectively protect plants against various environmental stresses, including salinity stress [31]. Rhizobacteria use tryptophan and other small molecules in root exudates and convert them into indole-3-acetic acid, which is utilized by the plant roots and resulted in the activation of plant’s endogenous auxin signaling pathway, which contribute in the growth promotion and proliferation of plant cells [32]. IAA accumulation in plants induces the transcription of ACC synthase genes, which increases the ACC concentration, leading to the production of ethylene. PGPR containing ACC deaminase may break down some of the excess ACC and lower plant ethylene levels during the advent of environmental stress [33].

Within this context, this study was designed to isolate ACC deaminase and indole-3-acetic acid (IAA) producing halotolerant PGPR because the survival and adaptation of PGPR to stressful environments are important to understand and confirm the survival of bacteria in real high-saline habitats. Moreover, the current study focused on investigating the various physio-chemical responses of tomato (*Solanum lycopersicum* L.) with the intention to explain the regulatory networks involved in osmotic stress tolerance and enhanced salinity stress tolerance following inoculation with ACC deaminase- and IAA-producing halotolerant PGPR.

## Results

### Isolation, selection and identification of *Leclercia adecarboxylata* MO1

**Fig. 1:**
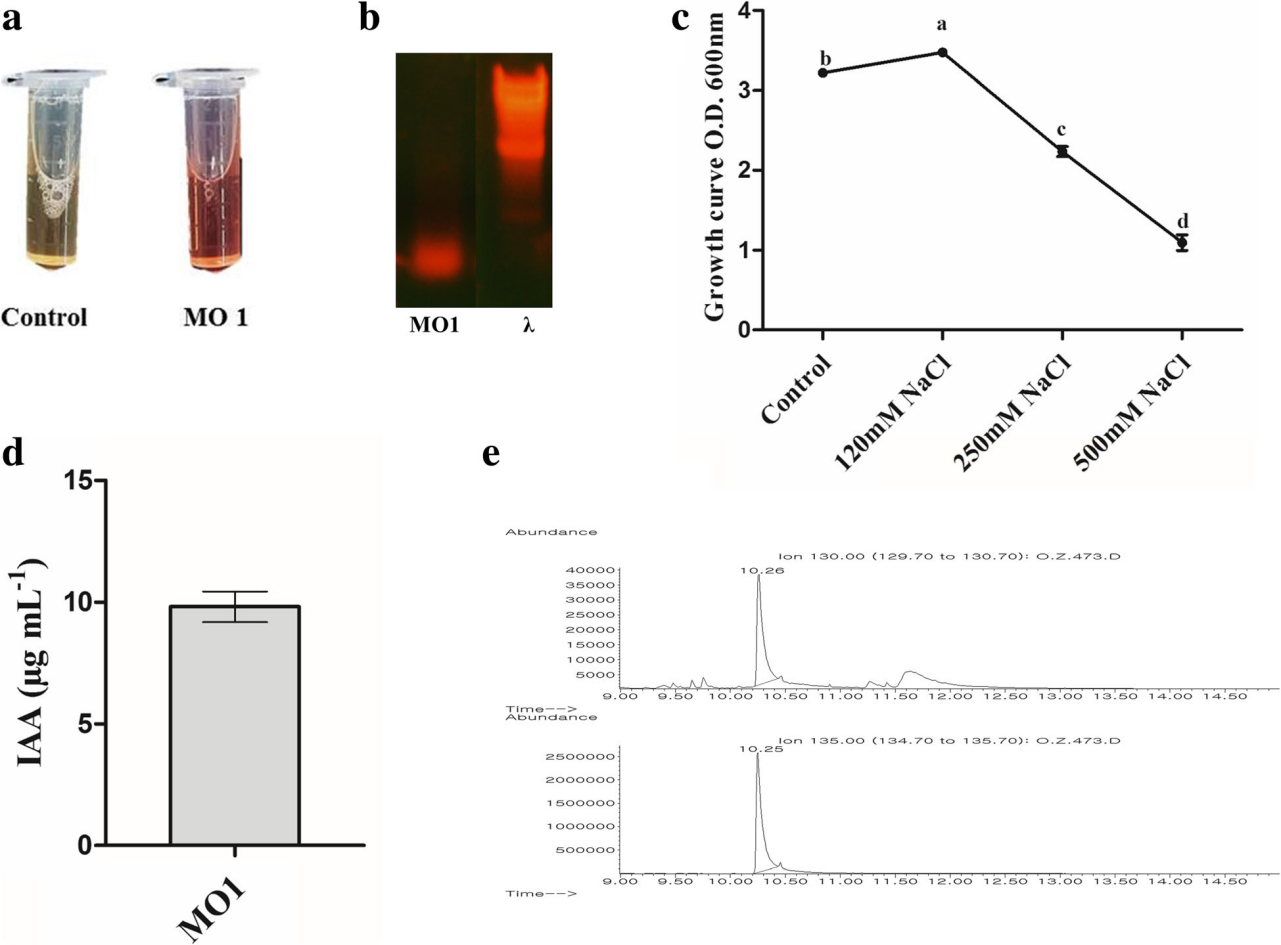
Isolation and selection of MO1. (**a**) Salkowski test for IAA (**b**) The presence of ACC deaminase responsible *acdS* gene (**c**) Salinity stress tolerance of MO1 (**d**) The amount of IAA produced by *L. adecarboxylata* MO1 (**e**) GC–MS/SIM spectrometry analysis of IAA produced by *L. adecarboxylata* MO1

The soil samples were collected from the rhizosphere of tomato (*Solanum lycopersicum* L.) plant at vegetative growth stage. The collected soil samples (10 g) were transferred to a 250 mL flask containing 100 mL Amies solution and then serially diluted to 1 × 10^− 4^. Then, a 0.1 mL suspension was spread on tryptic soy agar plates (TSA; Merck Co., Germany) and incubated at 28 °C for 24 h. The incubated plates were examined every 6 h to assess bacterial growth, and the newly appeared bacteria were re-streaked on new plates to get obtain colonies. Among total 36 isolated strains, *Leclercia adecarboxylata* MO1 was successfully isolated and selected on its IAA-producing capability and its ACC deaminase synthesis gene based on the Salkowski test and PCR results (Fig. 1). In addition, MO1 was grown under controlled conditions and under treatments with 120, 250, and 500 mM NaCl to examine its growth. The results showed that MO1 had maximal growth with a cell density of 3.47 ± 0.027 at OD_600_ in medium supplemented with 120 mM NaCl compared to that under control conditions, with a cell density of 3.21 ± 0.031. However, the higher concentrations (250 and 500 mM NaCl) negatively affected the growth of MO1, resulting in cell densities of 2.23 ± 0.064 and 1.09 ± 0.097 at OD_600_, respectively (Fig. 1).

**Fig. 2:**
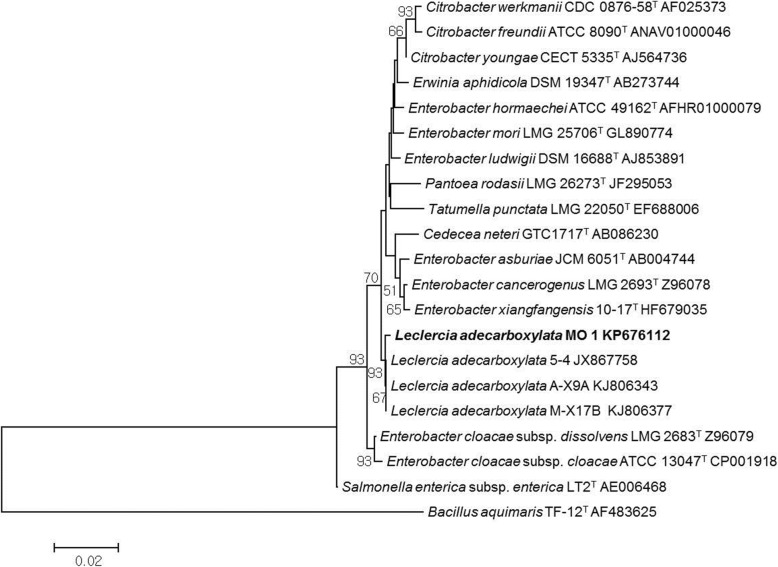
Phylogenetic analysis of bacterial isolate MO1 by MEGA 6. A neighbor-joining tree derived from aligning the most-similar 16S rRNA sequences in related taxa

Furthermore, MO1 was identified by 16S rRNA sequencing. An NCBI (http://www.ncbi.nlm.nih.gov/) BLAST search of the 16S rRNA sequence revealed that MO1 is closely related to the genus *Leclercia.* To further confirm this finding, a detailed phylogenetic analysis (MEGA 6.0) was performed, closely related sequences were aligned, and a neighbor-joining tree was constructed using MEGA 6.0 (Fig. 2). The MO1 showed high sequence homology and formed a subclade with *Leclercia adecarboxylata*. Based on these results, MO1 was identified as *L. adecarboxylata*, and its 16S rRNA sequence was submitted to the NCBI gene bank under the accession number KP676112.

### IAA production by MO1

The presence of IAA produced by MO1 in the inoculated cultural broth was initially detected with the Salkowski reagent and was further quantified by GC/MS (Fig. 1). The quantification results revealed that a significant amount of IAA (9.815 ± 0.6293 μg/mL) was produced by MO1 (Fig. 1).

### Effects of MO1 inoculation on plant growth and stress mitigation

**Fig. 3:**
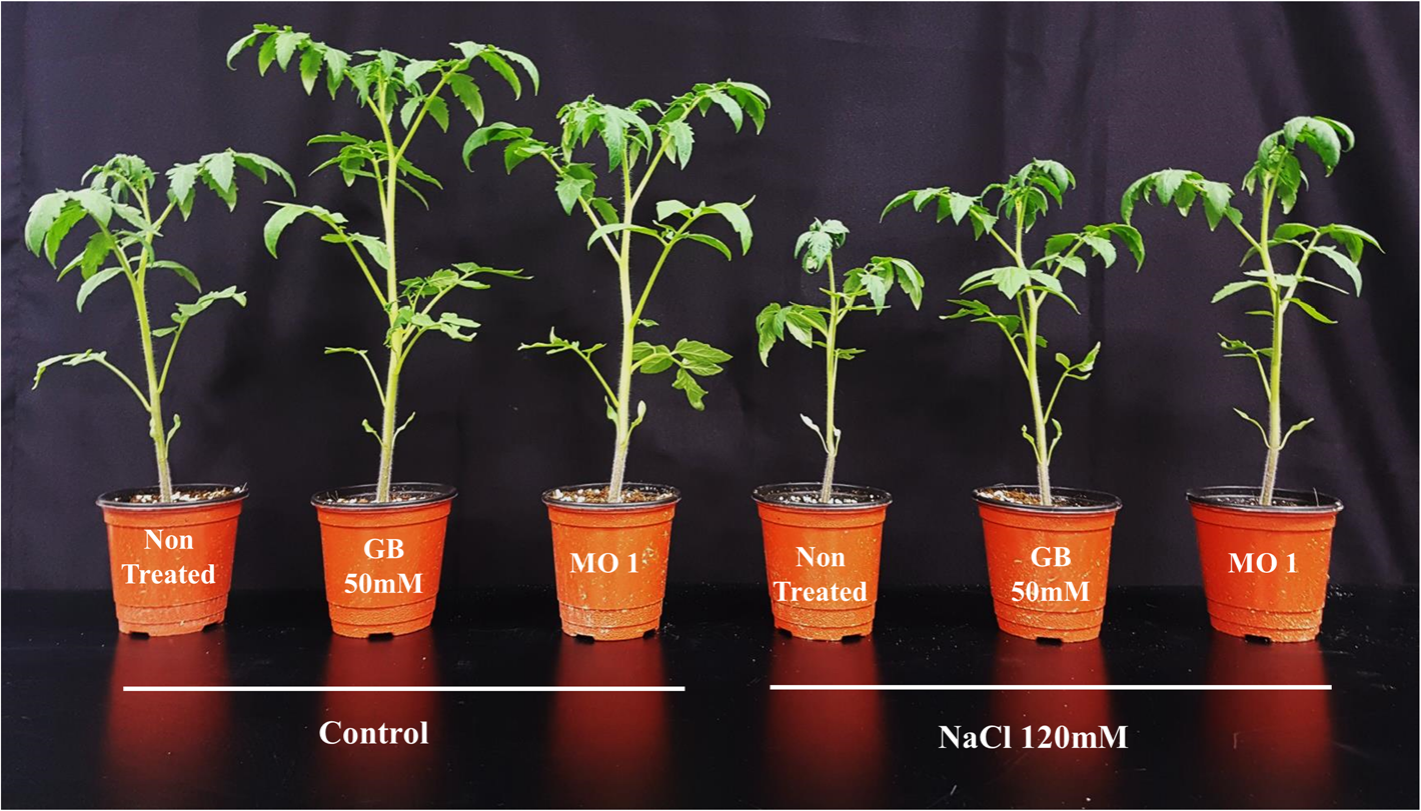
Plant growth-promoting potential of MO1 inoculation and GB treatment under normal and salinity stress conditions

The phytobeneficial and salinity stress-mitigating efficiency of MO1 were assessed by a plant microbe-interaction experiment using GB as positive and water as negative control. The results showed the positive correlation of MO1 with plant. The MO1 inoculation and GB treatment produced a fundamentally beneficial response under normal and stress conditions, while the NaCl treatment resulted in a hindered and lower growth potential (Fig. 3).

MO1 inoculation and GB treatment significantly improved all growth attributes, including shoot length, with 22.09 and 18% increases; root length, with 16.3 and 12.5% increases; shoot weight, with 28.01 and 27.22% increases; root weight, with 51.15 and 47.33% increases; and stem diameter, with 15.39 and 6.44% increases, respectively (Table 1).

**Table 1:** Effects of *L. adecarboxylata* MO1 inoculation and GB treatment on growth attributes of plants under normal and saline conditions

	Shoot length (cm)	Root length (cm)	Shoot fresh weight (g)	Root fresh weight (g)	Stem diameters (mm)
No Stress					
No treatment	23.90 ± 0.58b	14.72 ± 0.37b	10.14 ± 0.65b	1.31 ± 0.11b	4.66 ± 0.61a
MO1	29.18 ± 0.78a	17.12 ± 0.45a	12.98 ± 0.29a	1.98 ± 0.30a	5.39 ± 0.23a
50 mM GB	28.24 ± 0.54a	16.56 ± 0.40a	12.90 ± 0.45a	1.93 ± 0.15a	4.96 ± 0.11a
Salt Stress					
120 mM NaCl	19.28 ± 0.83b	13.38 ± 0.50c	5.53 ± 1.35c	0.81 ± 0.15b	3.66 ± 0.11b
MO1	26.94 ± 0.89a	16.24 ± 0.40a	9.44 ± 0.46a	1.48 ± 0.06a	4.43 ± 0.07a
50 mM GB	25.34 ± 1.16a	15.44 ± 0.34b	7.89 ± 0.62b	1.26 ± 0.22a	4.21 ± 0.14a

Values in columns followed by different letters are significantly different at *P* ≤ 0.05 among treatments based on DMRT

Moreover, salinity stress decreased plant growth attributes, while the MO1 inoculation and GB treatment significantly protected the plant against salinity stress (Fig. 3). Increases in shoot (39.83 and 31.43%), root weight (82.72 and 55.56%), and stem diameter (21.04 and 15.03%) were recorded in MO1-inoculated and GB-treated plants. Root length and shoot weight were significantly higher in MO1-inoculated plants, with 21.38 and 70.71% increases, followed by GB treatment, with 15.4 and 42.68% increases, respectively, compared to values in non-treated plants under saline conditions (Table 1).

### Influence of MO1 inoculation and GB treatment on chlorophyll fluorescence

**Fig. 4:**
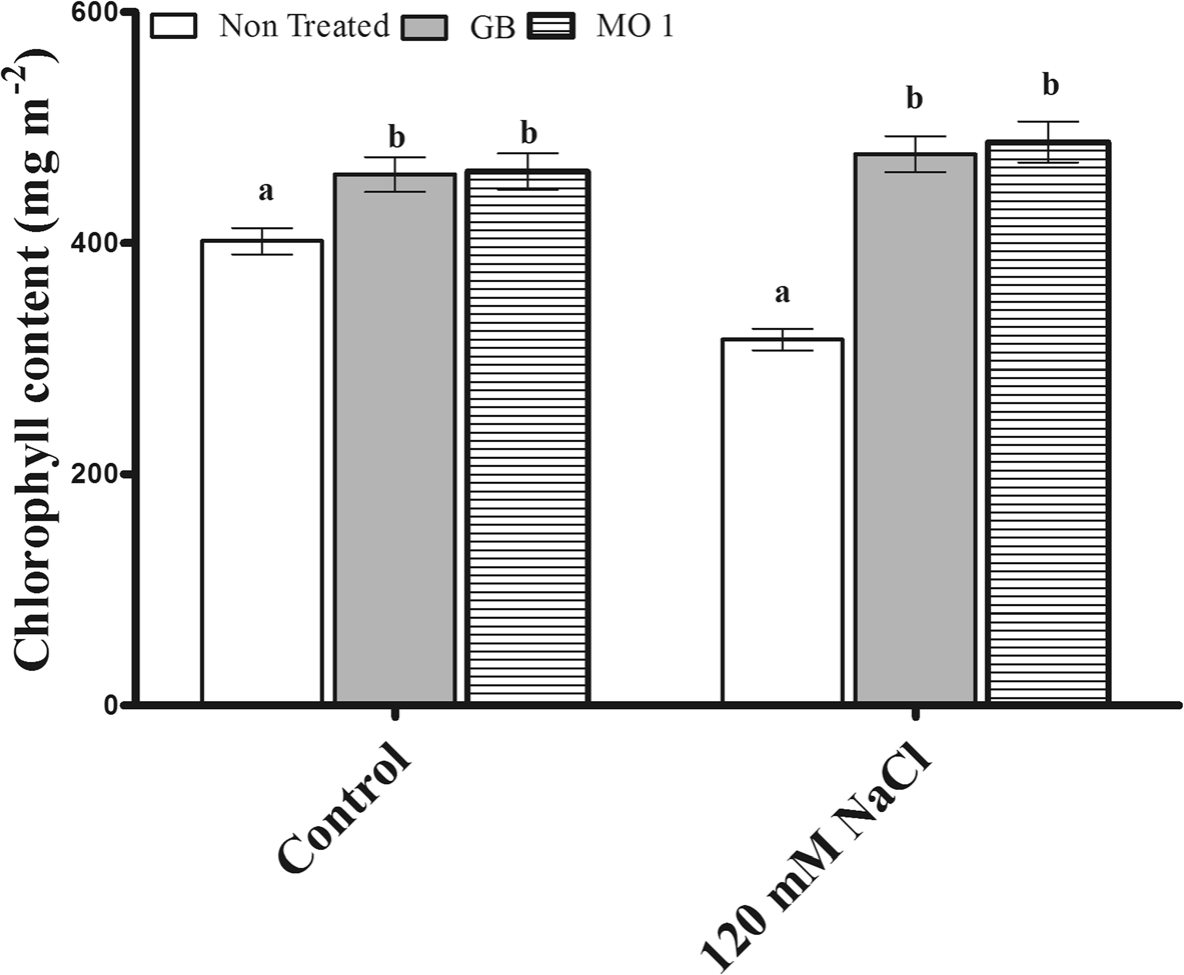
Effects of *L. adecarboxylata* MO1 inoculation and GB treatment on the chlorophyll fluorescence (Fv/Fm) of plants under normal and salinity stress conditions. Bars with different letters indicate significant differences (*P* ≤ 0.05) among treatments based on DMRT. Each value represents the mean ± SD of six replicates from each of three independent experiments

The regular exposure of plants to salinity stress suppresses and negatively regulates the chlorophyll contents. In the current study, MO1 inoculation and GB treatment significantly improved the chlorophyll fluorescence (Fv/Fm) of plants as compared to control (Fig. 4). Under normal condition, MO1 inoculation and GB treatment resulted in a higher chlorophyll fluorescence, with 15.03 and 14.39% increases, respectively, compared to that in the control (Fig. 4). Under salinity stress, a similar trend of improved chlorophyll fluorescence following MO1 inoculation and GB treatment was observed. A higher chlorophyll fluorescence was noted in MO1-inoculated and GB-treated plants, with 54.11 and 50.82% increases, respectively, compared to non-treated plants (Fig. 4).

### Sugar synthesis in response to MO1 inoculation and GB treatment during salinity stress

**Fig. 5:**
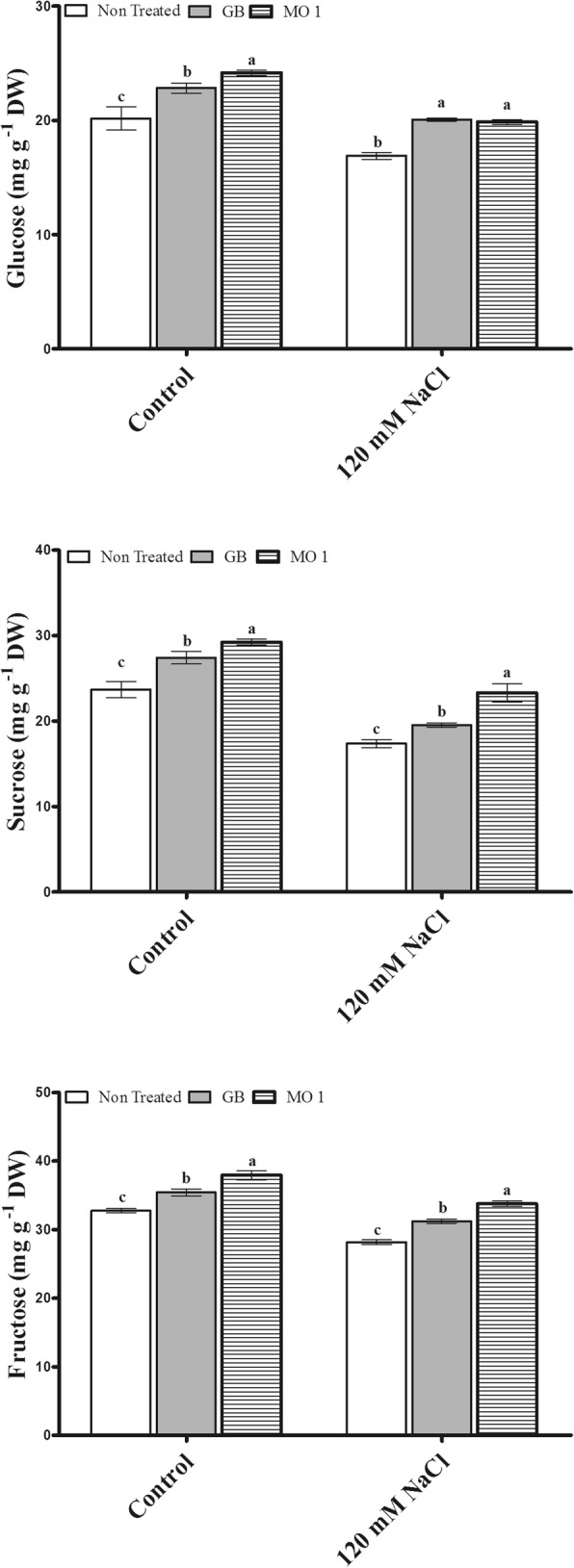
Sugar (glucose, sucrose, and fructose) regulation in response to *L. adecarboxylata* MO1 inoculation and GB treatment under normal and saline conditions. Bars with different letters indicate significant differences (*P* ≤ 0.05) among treatments based on DMRT. Each value represents the mean ± SD of six replicates from each of three independent experiments

In current study, the MO1 and GB treatments enhanced the glucose, sucrose, and fructose levels in plants grown under normal conditions compared to non-treated plants, while a negative trend of reduced glucose, fructose, and sucrose levels was observed in plants grown under saline conditions (Fig. 5). The changes in glucose, sucrose, and fructose levels in MO1-inoculated plants represented 19.83, 23.43, and 15.79% increases, respectively, followed by increases in GB-treated plants of 13.19, 15.75, and 8.18%, respectively, compared with the levels in non-treated plants under control conditions (Fig. 5).

Moreover, under saline conditions, glucose increased by 18.76 and 17.57% in GB-treated and MO1-inoculated plants, respectively, compared with the levels in non-treated plants (Fig. 5). The sucrose and fructose levels significantly increased following MO1 inoculation by 34.2 and 19.9%, respectively, and following GB treatment by 12.49 and 10.9%, respectively, compared to the levels in non-treated plants grown under salinity stress (Fig. 5).

### Organic acid regulation by MO1 inoculation and GB treatment under salinity stress

**Fig. 6:**
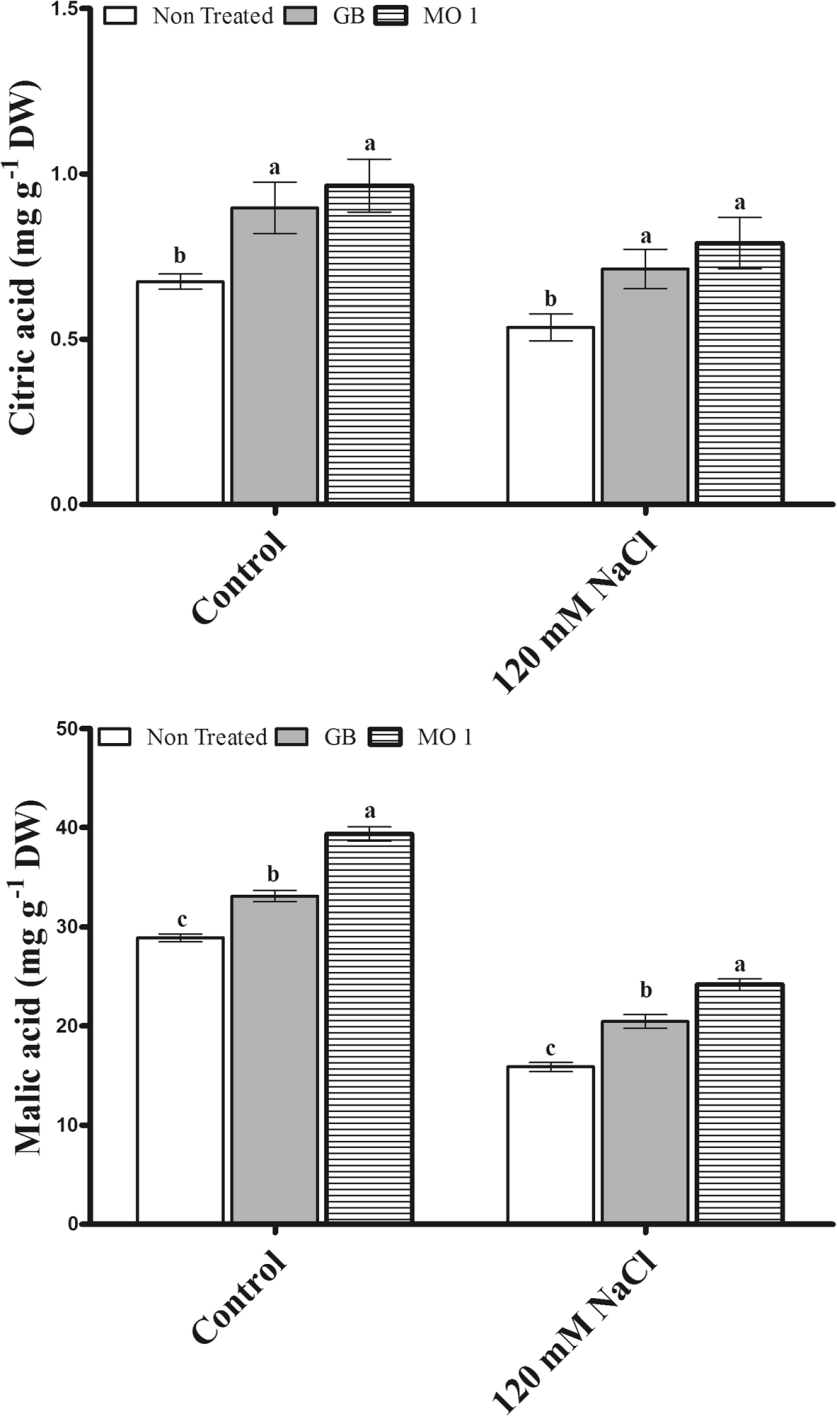
Organic acid regulation resulting from *L. adecarboxylata* MO1 inoculation and GB treatment under normal and saline conditions. Bars with different letters indicate significant differences (*P* ≤ 0.05) among treatments based on DMRT. Each value represents the mean ± SD of six replicates from each of three independent experiments

Organic acid contents (citric acid and malic acid) were significantly modulated following MO1 inoculation and GB treatment under normal and salinity stress conditions (Fig. 6). The results revealed significantly increased amounts of citric acid (43.26 and 33.14%) in MO1-inoculated and GB-treated plants, respectively, under control conditions (Fig. 6). Malic acid significantly increased by 36.18% following MO1 inoculation and by 14.48% following GB treatment compared to non-treated plants (Fig. 6).

A similar trend of differentially regulated citric and malic acid was found under salinity stress. The MO1 and GB treatments significantly increased the organic acid contents of plants under salinity stress (Fig. 6). The results revealed that the MO1 and GB treatments significantly increased the citric acid level, with 47.48 and 34.57% increases, respectively, compared to that in non-treated plants (Fig. 6). Moreover, a significant increase in malic acid of 52.19% was observed in MO1-inoculated plants followed by a 28.38% increase with GB treatment compared with that in non-treated plants (Fig. 6).

### Amino acid production in response to MO1 inoculation and GB treatment under salinity stress

**Fig. 7:**
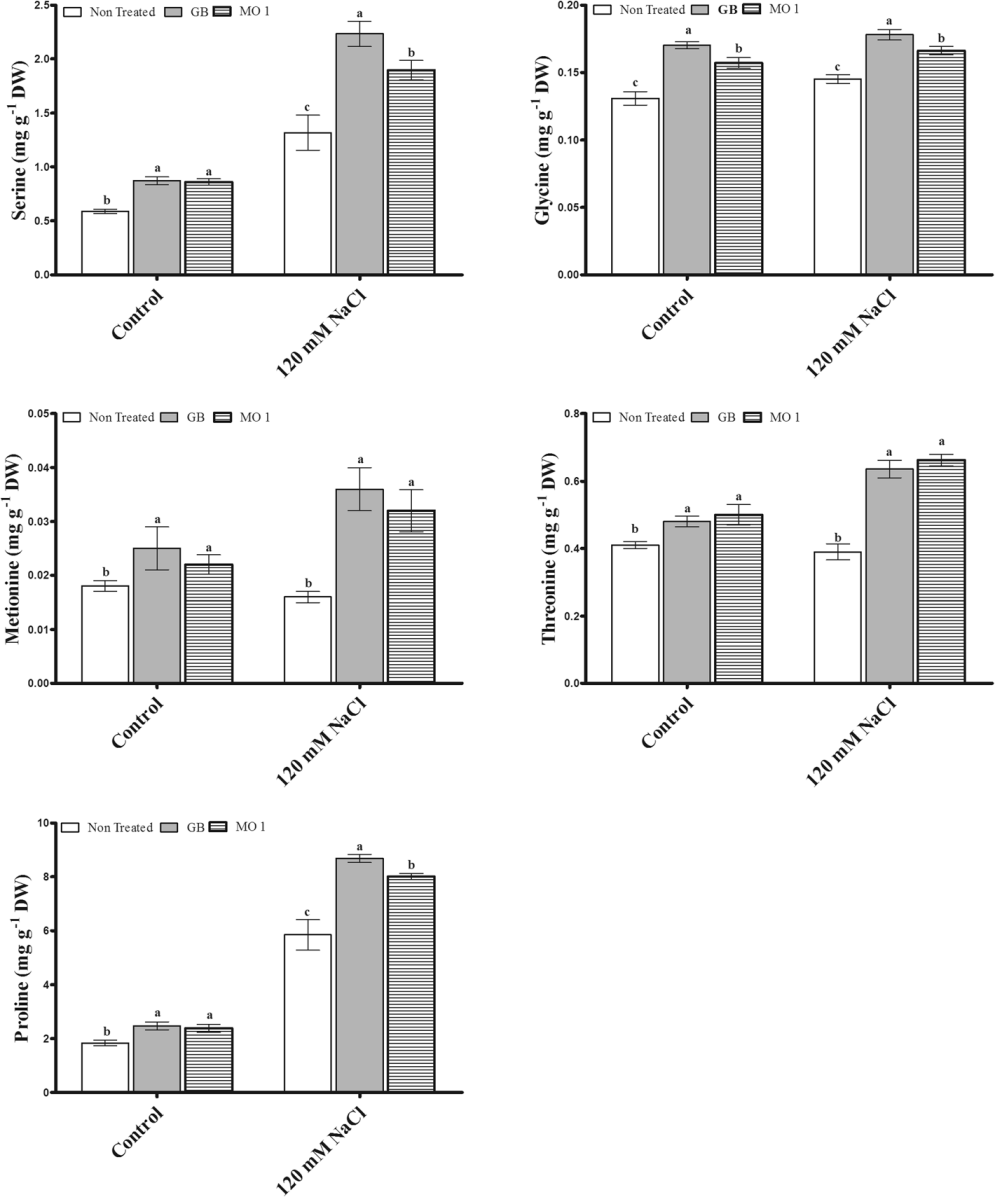
Amino acid regulation by *L. adecarboxylata* MO1 and GB treatment in plants under normal and saline conditions. Bars with different letters indicate significant differences (*P* ≤ 0.05) among treatments based on DMRT. Each value represents the mean ± SD of six replicates from each of three independent experiments

Amino acid levels were significantly changed in plants treated with and without MO1 and GB under normal and salinity stress conditions (Fig. 7). Under normal conditions, GB treatment and MO1 inoculation significantly up-regulated serine (48.55 and 46.5%), methionine (38.89 and 22.22%), glycine (29.77 and 19.85%), threonine (17.07 and 21.95%), and proline (34.68 and 29.61%), respectively, compared to levels in non-treated plants (Fig. 7).

Under saline conditions, higher amounts of serine, glycine, methionine, and proline were found in GB-treated plants, with 69.42, 22.76, 124.99, and 48.38% increases, respectively, followed by MO1-inoculated plants, with 43.78, 14.48, 100, and 36.92% increases, respectively, compared to levels in non-treated plants (Fig. 7). Significantly enhanced amount of threonine (70%) was recorded in MO1-inoculated plants followed by GB treatment (63.08%) compared to control (Fig. 7).

### Endogenous ABA modulation by MO1 and GB under stress

**Fig. 8:**
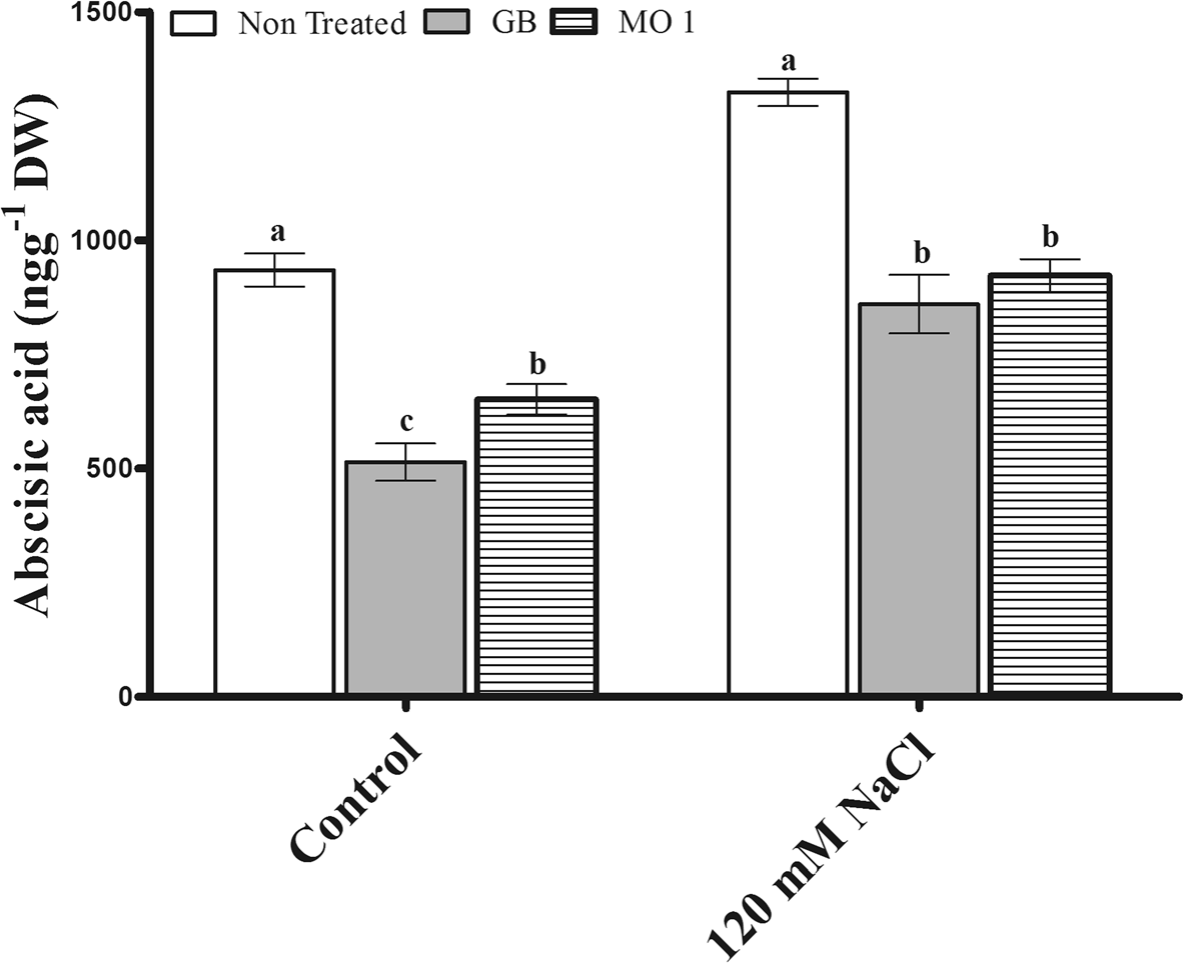
Influence of *L. adecarboxylata* MO1 inoculation and GB treatment on endogenous ABA levels in plants under normal and saline conditions. Bars with different letters indicate significant differences (*P* ≤ 0.05) among treatments based on DMRT. Each value represents the mean ± SD of six replicates from each of three independent experiments

Stress-responsive endogenous ABA was notably modulated in MO1-inoculated and GB-treated plants (Fig. 8). The results indicated that under control conditions, GB treatment significantly decreased the endogenous ABA level by 45% and that MO1 inoculation decreased it by 30.28% compared to the level in non-treated plants (Fig. 8).

The salinity stress exposure increased the endogenous ABA level in plants, while the MO1 inoculation and GB treatment significantly reduced the endogenous ABA level by 30.04 and 35.35%, respectively, compared to non-treated plants (Fig. 8).

## Discussion

The utilization of plant growth-promoting microbes for stress tolerance is an ideal and eco-friendly strategy [29]. To date, several studies have been carried out, in which PGPR were utilized for their potent role in salinity stress mitigation [27, 34–37]. Thus, the current study was intended to isolate an halotolerant IAA-producing bacteria and assess its potential to promote tomato (*Solanum lycopersicum* L.) growth and tolerance against salinity stress, and the selected isolate was identified as *L. adecarboxylata* MO1 (Figs. 1 and 2). *L. adecarboxylata*, formerly known as *Escherichia adecarboxylata*, belongs to the *Enterobacteriaceae* family [38]. Because of electrophoretic and nucleic differences, the *E. adecarboxylata* was separated from the *Enterobacter agglomerans* and reclassified as *L. adecarboxylata* [39]. *L. adecarboxylata* is widely distributed in nature and has been isolated from various sources, including seeds, plants, rhizosphere, food, water, and various other environmental sources [38–42].

*L. adecarboxylata* is metabolically diverse and can produce phytohormones, synthesize extra-cellular enzymes, degrade hydrocarbons, and solubilize minerals [40, 43–45]. Such traits have been reported to improve plant growth and mitigate various stresses [46]. Enzymes include ACC (1-aminocyclopropane-1-carboxylic acid) daeminase are related with free living rhizobacteria, which play an important role in facilitating plant growth [47]. In current study *L. adecarboxylata* MO1 showed the presence of genes responsible for the deamination of ACC (Fig. 1). ACC, the immediate precursor for ethylene synthesis in plants is exuded from roots of plants, which is metabolized by bacteria having the potential to produce ACC deaminase [48]. ACC deaminase catalyzes the conversion of ACC to ammonia and α-ketobutyrate [49]. This conversion lower the ethylene level in plants and resulted in plant growth promotion. Moreover, there is much less ethylene in the presence of ACC deaminase and consequent ethylene feedback inhibition of IAA signal transduction, so that bacteria IAA can continue to promote the growth and increase ACC synthase transcription. This IAA and ACC deaminase cross-talking resulted in lowering the ethylene level and revealed that ACC deaminase facilitate the plant growth by IAA [47]. The IAA-producing potential of *L. adecarboxylata* MO1 (Fig. 1) is in line with that reported by Shahzad et al. [40]. Moreover, the growth-promoting of capability of *L. adecarboxylata* has been widely reported [50]; however, the role of *L. adecarboxylata* in stress mitigation is not well understood, making the proper investigation of its potent role in mitigating stress and improving stress tolerance necessary. The current study is the first report on the salinity stress mitigation potential of *L. adecarboxylata.*

In our study, salinity stress caused a drastic decrease in the chlorophyll fluorescence (Fv/Fm) of plants. However, the MO1 inoculation and GB treatment increased the chlorophyll fluorescence (Fig. 4). The increase in chlorophyll fluorescence under normal and stress conditions following *L. adecarboxylata* inoculation and GB treatment might be linked to the enhanced pigment synthesis [51]. Moreover, during chlorophyll synthesis, plants fix the carbon dioxide into sugar by using sunlight [52]. Sugar (glucose, sucrose, and fructose) is an important source of energy to stimulate plant growth and survival under normal and stress conditions [53, 54]. High compatible sugar contents play an important role in salinity stress mitigation and enhanced plant tolerance. In the current study, *L. adecarboxylata* MO1 inoculation and GB treatment significantly increased the sugar (glucose, sucrose, and fructose) contents in plants grown under normal and salinity stress conditions, while a reduced level of soluble sugar in plants under stress condition, suggesting the positive role of *L. adecarboxylata* MO1 and GB in salinity stress (Fig. 5). Under saline conditions, the thylakoid membranes are damaged, which results in reduced photosynthetic efficiency and less soluble sugar production [51].

Phytobeneficial microorganisms can stimulate organic acid metabolism in plants under stress conditions [55, 56]. Organic acids play an important role as a solute in osmatic adjustment and excess cation balance under salinity stress [55]. In the current study, the MO1 inoculation and GB treatment significantly increased organic acids under normal as well as under salinity stress conditions (Fig. 6). Similar results of increases in organic acids following inoculation with plant growth-promoting bacteria under osmotic stress have been reported [55, 57]. Organic acids also help plants cope with nutrient deficiencies and stress tolerance because they have the potent ability to displace phosphorus from insoluble complexes and make it available for uptake by plants [58]. Moreover, organic acids have also been reported to be involved in plant microbe interactions at the root/soil interface, as organic acids secreted by roots are known to act in chemical signaling for quorum sensing, exopolysaccharide secretion, and biofilm formation during rhizosphere colonization [59, 60].

Amino acids are also used as either precursors or intermediates for important metabolites responsible for mitigating various biotic and abiotic stresses [61, 62]. In addition, plant growth-promoting microbes also secrete amino acids in soil, and plants absorb them through their roots via important mechanisms [63, 65]. The increased levels of amino acids in MO1-inoculated and GB-treated plants under normal and salinity stress conditions (Fig. 7) reflected their role in strengthening plant tolerance against salinity stress by various mechanisms [24].

In the current study, the endogenous ABA level was significantly reduced by MO1 inoculation and GB treatment (Fig. 8). The decrease in the ABA level revealed positive correlation between MO1 and GB and plant growth and stress tolerance. As ABA is involved in stress signaling, its biosynthesis can be affected during plant growth-promoting microbe interactions [24, 64]. Several studies have reported increases in ABA under salinity and osmotic stresses. However, many plant growth-promoting microbes have been reported to reduce ABA accumulation under stress conditions [24, 37, 65]. However, some microorganisms have also been reported to increase the endogenous ABA level, but the effect depends on the various classes of microorganisms [66]. The reduction of ABA accumulation in MO1-inoculated plants is correlated with its IAA-producing capability, resulting in improved plant growth and improved water relation [67], suggesting an active role of PGPRB in stress resistance.

## Conclusion

Salinity stress is one the major factors that hinder agriculture productivity, and the development of stress-resistant varieties via breeding and genetic engineering is a lengthy and expensive process. However, the utilization of plant growth-promoting microbes to alleviate stress is a more cost-effective and environmentally friendly approach. Delivery of ACC-deaminase and IAA via PGPR mostly affected ethylene and ABA-dependent signaling in positive way which facilitate plant growth and mitigate stressful condition positively [47]. The results of our study indicated that halotolerant *L. adecarboxylata* MO1 could reprogram plants under salinity stress to improve their growth and provide resistance via ACC deaminase synthesis and IAA production which significantly modulate plants endogenous sugar, organic acids, amino acids and stress responsive ABA. These results will guide the necessary future studies for tomato cultivation in areas where salinity is a major constraint. However, further research is required to validate the effectiveness of this PGPR isolate before recommendation for large scale at the field level.

## Methods

### Isolation

Soil samples having T-N 0.171%, P_2_O_5_ 0.026%, K 0.029 ppm, Na 0.050 ppm, Ca 0.057 ppm and Mg 0.094 ppm were collected from Gyeongbuk province in South Korea for isolation of bacteria from rhizosphere of tomato according to the method described by Kang et al. [65]. Briefly, collected soil samples (10 g) were transferred to a 250 mL flask containing 100 mL Amies solution [69] and then serially diluted to 1 × 10^− 4^. Then, a 0.1 mL suspension was spread on tryptic soy agar plates (TSA; Merck Co., Germany) and incubated at 28 °C for 24 h. The incubated plates were examined every 6 h to assess bacterial growth, and the newly appeared bacteria were re-streaked on new plates to get obtain colonies. Single pure colonies were cultured in LB (Luria-Bertani) broth.

### Selection of effective isolate

The initial screening for halotolerant IAA and ACC deaminase producing bacteria was carried out according to the method described by Shahzad et al. [24]. Briefly, 1 mL Salkowski reagent was added to 1 mL culture filtrate of bacterial isolates. Among the various bacterial isolates, MO1 changed the color to pink, indicating that MO1 produced IAA [68].

Moreover, presence of ACC deaminase responsible gene ‘*acdS*’ were examined by PCR analysis using (5′–3′) primers (Forward: ATCGGCGGCATCCAGWSNAAYCANAC and Reverse: GTGCATCGACTTGCCCTCRTANACNGGRT) as describe by Wang et al. [70]. Briefly, PCR was carried out for 35 cycles with the initial denaturation at 94 °C for 3 min, cyclic denaturation at 94 °C for 30 s, annealing 58 °C for 30 s and extension at 72 °C for 2 min with a final extension of 7 min at 72 °C using 50 μL reaction mixture containing 50 ng of DNA, 20 pmoles of each primer, 1.25 units of Taq DNA polymerase, 200 μM of each dNTPs and 1× PCR buffer. Moreover the PCR product was examined by agarose gel electrophoresis.

In addition, the salinity tolerance potential of MO1 in a saline environment was investigated according to the method described by Shahzad et al. [24]. Briefly, *MO1* was grown in three concentrations of NaCl (120, 250, and 500 mM) in LB media (10 g tryptone, 5 g yeast extract, 10 g NaCl, pH 7.0 ± 0.2, autoclaved for 15 min at 121 °C) to determine its growth dynamics and salinity tolerance capability. Growth dynamics were assessed using bacterial cell density at OD_600_ (T60 UV VIS Spectrophotometer).

### Phylogenetic analysis

The MO1 bacterial strain was identified by PCR amplification and sequencing of 16S rRNA and phylogenetic analysis. The specific 27F primer (5′-AGAGTTTGATC (AC) TGGCTCAG-3′) and 1492R primer (5′-CGG (CT) TACCTTGTTACGACTT-3′) were used for PCR as described by Shahzad et al. [71].

### IAA quantification by GC/MS

IAA produced by MO1 in culture broth without L-Trp was extracted and analyzed by GC/MS with selected ion monitoring (SIM; 6890 N network GC system and 5973 network mass selective detector; Agilent Technologies, Santa Clara, CA, USA). The GC/MS conditions used for IAA quantification are given in the supplementary table (Additional file 1: Table S1) according to the method described by Shahzad et al. [40].

### Salinity stress application and PGPR inoculation

A plant microbe interaction experiment was carried out to confirm the growth-promoting and stress-mitigating potential of the isolated *L. adecarboxylata* MOI. The experiment was carried out in complete randomized design with six treatments (Control, MO1, and GB each with and without 120 mM NaCl) in triplicate. Tomato (*Solanum lycopersicum* L. ‘Yegwang’) seeds were purchased from Danong Co. (Korea) and surface sterilized with 70% EtoH followed by 2.5% NaOH and rinsed with deionized distilled water and were germinated at 28 °C in an incubator. Equal-sized seedlings (1 seedling per pot) were moved to sterilized plastic pots (10 cm width and 9 cm height) filled with 500 mg autoclaved horticultural substrate composed of peat moss (10–15%), coco peat (45–50%), perlite (35–40%), and zeolite (6–8%) with the NO_3_^−^ (∼0.205 mg g^− 1^), NH^4+^ (∼0.09 mg g^− 1^), P_2_O_5_ (∼0.35 mg g^− 1^), and K_2_O (∼0.1 mg g ^− 1^). Moreover, the soil pH was 5–7, bulk density was under 0.3 mg/m^3^, and EC (dS/m) was ≤1.2 [19]. The pots were placed in a growth chamber with a fixed program (day/night cycle: 12 h at 24 °C, 12 h at 20 °C; relative humidity: 65–70%; 1000 μE m − 2 s − 1 from sodium lamps). After two weeks, the pots were divided into six group according to the experimental design with 30 plants per treatment in triplicate. The plants were inoculated with 20 mL MO1 (4 × 10^8^ cells mL^− 1^) and treated with GB followed by 50 mL 120 mM NaCl for 10 days on a daily basis to induce salinity-induced osmotic stress [72, 73]. Upon stress completion (10 days) at vegetative growth stage, growth attributes (shoot and root length, shoot and root fresh weight, stem diameter, and chlorophyll content) were recorded and the plants were immediately harvested in liquid nitrogen and stored in − 80 °C until further biochemical analyses.

### Chlorophyll content assessment

Chlorophyll content was examined using a CCM-300 Chlorophyll Content Meter (Opti-Sciences, Inc., Hudson, NH, USA) as previously described by Kim et al. [74].

### Sugar quantification

The contents of soluble sugars such as glucose, sucrose, and fructose were estimated according to the method described by Khan et al. [75]. Briefly, 0.5 g freeze dried grinded samples were extracted with 80% ethanol followed by vacuum drying. The dried residue was re-dissolved in 1 mL deionized water and passed through 0.45 μm Nylon-66 syringe filters. Furthermore, the filtered samples were injected to HPLC (Millipore Co., Waters Chromatography, Milford, MA, USA).

### Organic acid estimation

Organic acids were measured according to the method described by Bilal et al. [76] . Briefly, 0.1 g of freeze dried grinded samples were added to 9 mL of distilled water and left overnight at room temperature. Then the sample was passed through a 0.22 μm syringe filter and 20 μL using HPLC on a model 600E system (Waters, Millford, MA, USA) equipped with a refractive index detector (RI, Model 410) with fixed isocratic conditions (mobile phase: 0.005 M H_2_SO_4_ in water, flow rate 0.6 mL min^− 1^, column temperature 65 °C, PL Hi-Plex H column).

### Amino acid determination

Amino acids were determined according to the method described by Shahzad et al. [77]. Briefly, freeze dried plant samples were hydrolyzed in 6 N HCl under vacuum in 4 mL tubes at 110 °C for 24 h, followed by 80 °C for 24 h. The dried residue was homogenized in 0.02 N HCl and was passed through 0.45-μm filter. Amino acids were quantified by an automatic amino acid analyzer (HITACHI L-8900, Japan) attached to a HITACHI HPLC system (packed column with ion-exchanging resin, No. 2622 PF; 4.6 × 60 mm) and ultraviolet detector (VIS1: 570 nm, VIS2: 440 nm).

### ABA quantification

ABA was analyzed by GCMS (6890 N network GC system and 5973 network mass selective detector, Agilent Technologies, Palo Alto, CA, USA) according to the method described by Qi et al. [78]. Moreover, [(±)-3,5,5,7,7,7-d^6^]-ABA was added as internal standard and for quantification, the Lab-Base (ThermoQuset, Manchester, UK) data system software was used to monitor responses to ions of m/e 162 and 190 for Me-ABA Me-[^2^H_6_]-ABA and *m/z* 166 and 194 for Me-[2H6]-ABA.

### Statistical analysis

The data which are presented as means ± standard deviation were collected and pooled from triplicate treatments from each of three independently repeated experiments and were subjected to Duncan’s multiple range test (DMRT) using SAS version 9.2 (Cary, NC, USA). Graphs were constructed using GraphPad prism. Moreover, the biochemical data obtained from each repetition were pooled together for their respective treatment and grouped into normal and stress conditions. A two-way analysis of variance (ANOVA) was performed to determine the significance level (*P* < 0.05). The two way ANOVA table is given in the Additional file 2: Table S2.

## Additional files

Additional file 1: **Table S1.** GC/MS – SIM conditions used for analysis and quantification of the indole-3-acetic acid. (DOCX 12 kb)

Additional file 2: **Table S2.** Two-way ANOVA table of the biochemical analysis performed for tomato plants with and without MO1 inoculation and GB treatment under normal and 120 mM NaCl stress. (DOCX 18 kb)

## Abbreviations

ABA: Abscisic acid
GA: Gibberellins.
GB: Glycine betaine
IAA: Indole-3-acetic acid

## References

[1] Pereira A (2016). Plant abiotic stress challenges from the changing environment. Front Plant Sci.

[2] Shabala S, Munns R (2017). Salinity stress: physiological constraints and adaptive mechanisms. Plant Stress Physiol.

[3] Cheeseman J. Food security in the face of salinity, drought, climate change, and population growth: Halophytes for Food Security in Dry Lands. Elsevier; 2016. p. 111–23.

[4] Dagar JC (2016). Agroforestry for the Management of Waterlogged Saline Soils and Poor- quality Waters Advances in Agroforestry.

[5] Zhu J-K (2001). Plant salt tolerance. Trends Plant Sci.

[6] Hussain MI, Al-Dakheel AJ, Reigosa MJ (2018). Genotypic differences in agro-physiological, biochemical and isotopic responses to salinity stress in quinoa (*Chenopodium quinoa* Willd.) plants: prospects for salinity tolerance and yield stability. Plant Physiol Biochem.

[7] Petropoulos SA, Levizou E, Ntatsi G, Fernandes Â, Petrotos K, Akoumianakis K (2017). Salinity effect on nutritional value, chemical composition and bioactive compounds content of Cichorium spinosum L. Food Chem.

[8] Asrar H, Hussain T, Hadi SMS, Gul B, Nielsen BL, Khan MA (2017). Salinity induced changes in light harvesting and carbon assimilating complexes of Desmostachya bipinnata (L.) Staph. Environ Exp Bot.

[9] Ishikawa T, Shabala S. Control of xylem Na+ loading and transport to the shoot in rice and barley as a determinant of differential salinity stress tolerance. Physiol Plant. 2018. 10.1111/ppl.12758 29761494

[10] Garg N, Bhandari P (2016). Silicon nutrition and mycorrhizal inoculations improve growth, nutrient status, K+/Na+ ratio and yield of Cicer arietinum L. genotypes under salinity stress. Plant Growth Regul.

[11] Flowers TJ, Flowers SA (2005). Why does salinity pose such a difficult problem for plant breeders?. Agric Water Manag.

[12] Fan Y, Bose J, Zhou M, Shabala S. ROS production, scavenging, and signaling under salinity stress. Manag Salt Toler Plants Mol Genomic Perspect. 2015;187.

[13] Pang C-H, Wang B-S. Oxidative stress and salt tolerance in plants: Progress in botany. Springer; 2008. p. 231–45.

[14] Yurtseven E, Kesmez GD, Ünlükara A (2005). The effects of water salinity and potassium levels on yield, fruit quality and water consumption of a native central anatolian tomato species (*Lycopersicon esculantum*). Agric Water Manag.

[15] Singh J, Sastry ED, Singh V (2012). Effect of salinity on tomato (*Lycopersicon esculentum* mill.) during seed germination stage. Physiol Mol Biol Plants.

[16] La Pena RD, Hughes J (2007). Improving vegetable productivity in a variable and changing climate. Journal of Semi-Arid Tropical Agricultural Research.

[17] Wu M, Kubota C (2008). Effects of electrical conductivity of hydroponic nutrient solution on leaf gas exchange of five greenhouse tomato cultivars. HortTechnology..

[18] Sato S, Sakaguchi S, Furukawa H, Ikeda H (2006). Effects of NaCl application to hydroponic nutrient solution on fruit characteristics of tomato (Lycopersicon esculentum mill.). Sci Hortic.

[19] Shahzad R, Khan AL, Bilal S, Asaf S, Lee I-J. What is there in seeds? Vertically transmitted endophytic resources for sustainable improvement in plant growth. Front Plant Sci. 2018;9. 10.3389/fpls.2018.00024 PMC5787091 29410675

[20] Shameer S, Prasad T (2018). Plant growth promoting rhizobacteria for sustainable agricultural practices with special reference to biotic and abiotic stresses. Plant Growth Regul.

[21] Yang J, Kloepper JW, Ryu C-M (2009). Rhizosphere bacteria help plants tolerate abiotic stress. Trends Plant Sci.

[22] Mishra J, Fatima T, Arora NK. Role of Secondary Metabolites from Plant Growth-Promoting Rhizobacteria in Combating Salinity Stress. In: Plant Microbiome: Stress Response. Springer; 2018. p. 127–163.

[23] Fukami J, de la Osa C, Ollero FJ, Megías M, Hungria M (2018). Co-inoculation of maize with Azospirillum brasilense and rhizobium tropici as a strategy to mitigate salinity stress. Funct Plant Biol.

[24] Shahzad R, Khan AL, Bilal S, Waqas M, Kang S-M, Lee I-J. Inoculation of abscisic acid-producing endophytic bacteria enhances salinity stress tolerance in Oryza sativa. Environ Exp Bot. 2017;136.

[25] Kumar A, Singh VK, Tripathi V, Singh PP, Singh AK. Plant growth-promoting Rhizobacteria (PGPR): perspective in agriculture under biotic and abiotic stress: Crop Improvement Through Microbial Biotechnology. Elsevier; 2018. p. 333–42.

[26] Patel TS, Minocheherhomji FP (2018). Plant growth promoting Rhizobacteria: blessing to agriculture. Int J Pure App Biosci.

[27] Vimal SR, Patel VK, Singh JS. Plant growth promoting Curtobacterium albidum strain SRV4: an agriculturally important microbe to alleviate salinity stress in paddy plants. Ecol Indic. 2018.

[28] Sapre S, Gontia-Mishra I, Tiwari S (2018). Klebsiella sp. confers enhanced tolerance to salinity and plant growth promotion in oat seedlings (Avena sativa). Microbiol Res.

[29] Kumar K, Amaresan N, Madhuri K (2017). Alleviation of the adverse effect of salinity stress by inoculation of plant growth promoting rhizobacteria isolated from hot humid tropical climate. Ecol Eng.

[30] Fazal A, Bano A (2016). Role of plant growth-promoting rhizobacteria (pgpr), biochar, and chemical fertilizer under salinity stress. Commun Soil Sci Plant Anal.

[31] Latif Khan A, Ahmed Halo B, Elyassi A, Ali S, Al-Hosni K, Hussain J (2016). Indole acetic acid and ACC deaminase from endophytic bacteria improves the growth of solarium lycopersicum. Electron J Biotechnol.

[32] Dakora FD, Phillips DA (2002). Root exudates as mediators of mineral acquisition in low-nutrient environments. Food Security in Nutrient-Stressed Environments.

[33] Gamalero E, Glick BR (2015). Bacterial modulation of plant ethylene levels. Plant Physiol.

[34] Yousefi S, Kartoolinejad D, Bahmani M, Naghdi R (2017). Effect of Azospirillum lipoferum and Azotobacter chroococcum on germination and early growth of hopbush shrub (Dodonaea viscosa L.) under salinity stress. J Sustain For.

[35] Habib SH, Kausar H, Saud HM. Plant growth-promoting rhizobacteria enhance salinity stress tolerance in okra through ROS-scavenging enzymes. Biomed Res Int. 2016;2016. 10.1155/2016/6284547 PMC4756578 26951880

[36] Paulucci NS, Gallarato LA, Reguera YB, Vicario JC, Cesari AB, de Lema MBG (2015). Arachis hypogaea PGPR isolated from argentine soil modifies its lipids components in response to temperature and salinity. Microbiol Res.

[37] Kang S-M, Khan AL, Waqas M, You Y-H, Kim J-H, Kim J-G (2014). Plant growth-promoting rhizobacteria reduce adverse effects of salinity and osmotic stress by regulating phytohormones and antioxidants in Cucumis sativus. J Plant Interact.

[38] Richard C (1984). Nouvelles espèces de Enterobacteriaceae. Bull Inst Pasteur.

[39] Tamura K, Sakazaki R, Kosako Y, Yoshizaki E (1986). Leclercia adecarboxylata gen. Nov., comb. Nov., formerly known asEscherichia adecarboxylata. Curr Microbiol.

[40] Shahzad R, Waqas M, Khan AL, Al-Hosni K, Kang S-M, Seo C-W, et al. Indoleacetic acid production and plant growth promoting potential of bacterial endophytes isolated from rice (*Oryza sativa* L.) seeds. Acta Biol Hung. 2017;68. 10.1556/018.68.2017.2.5 28605980

[41] Verma P, Yadav AN, Khannam KS, Panjiar N, Kumar S, Saxena AK (2015). Assessment of genetic diversity and plant growth promoting attributes of psychrotolerant bacteria allied with wheat (Triticum aestivum) from the northern hills zone of India. Ann Microbiol.

[42] Kelemu S, Fory P, Zuleta C, Ricaurte J, Rao I, Lascano C (2011). Detecting bacterial endophytes in tropical grasses of the Brachiaria genus and determining their role in improving plant growth. African J Biotechnol.

[43] Sun K, Liu J, Gao Y, Jin L, Gu Y, Wang W (2014). Isolation, plant colonization potential, and phenanthrene degradation performance of the endophytic bacterium Pseudomonas sp. Ph6-gfp. Sci Rep.

[44] Pei-Xiang Y, Li MA, Ming-Hui C, Jia-Qin XI, Feng HE, Chang-Qun D (2012). Phosphate solubilizing ability and phylogenetic diversity of bacteria from P-rich soils around Dianchi Lake drainage area of China. Pedosphere..

[45] Sarma PM, Bhattacharya D, Krishnan S, Lal B (2004). Degradation of polycyclic aromatic hydrocarbons by a newly discovered enteric bacterium. Leclercia adecarboxylata Appl Environ Microbiol.

[46] Paul S, Dukare AS, Manjunatha BS, Annapurna K (2017). lant Growth-Promoting Rhizobacteria for Abiotic Stress Alleviation in Crop. Advances in Soil Microbiology.

[47] Glick BR (2014). Bacteria with ACC deaminase can promote plant growth and help to feed the world. Microbiol Res.

[48] Penrose DM, Moffatt BA, Glick BR (2001). Determination of 1-aminocycopropane-1-carboxylic acid (ACC) to assess the effects of ACC deaminase-containing bacteria on roots of canola seedlings. Can J Microbiol.

[49] Penrose DM, Glick BR (2003). Methods for isolating and characterizing ACC deaminase-containing plant growth-promoting rhizobacteria. Physiol Plant.

[50] Kisiel A, Kępczyńska E (2016). Medicago truncatula Gaertn. As a model for understanding the mechanism of growth promotion by bacteria from rhizosphere and nodules of alfalfa. Planta..

[51] Radhakrishnan R, Lee IJ (2014). Effect of low dose of spermidine on physiological changes in salt-stressed cucumber plants. Russ J Plant Physiol.

[52] Gupta AK, Kaur N (2005). Sugar signalling and gene expression in relation to carbohydrate metabolism under abiotic stresses in plants. J Biosci.

[53] Sami F, Yusuf M, Faizan M, Faraz A, Hayat S (2016). Role of sugars under abiotic stress. Plant Physiol Biochem.

[54] Lastdrager J, Hanson J, Smeekens S (2014). Sugar signals and the control of plant growth and development. J Exp Bot.

[55] Esringü A, Kaynar D, Turan M, Ercisli S (2016). Communications in Soil Science and Plant Analysis Ameliorative Effect of humic acid and plant growth-promoting Rhizobacteria ( PGPR ) on Hungarian vetch plants under salinity stress ameliorative effect of humic acid and plant growth-promoting. Commun Soil Sci Plant Anal.

[56] Lugtenberg BJ, Malfanova N, Kamilova F, Berg G. Plant Growth Promotion by Microbes. Molecular microbial ecology of the rhizosphere. 2013. 3;2:561–573.

[57] Cramer GR, Ergül A, Grimplet J, Tillett RL, Tattersall EAR, Bohlman MC (2007). Water and salinity stress in grapevines: early and late changes in transcript and metabolite profiles. Funct Integr Genomics.

[58] Richardson AE, Barea J, McNeill AM, Prigent-Combaret C. Acquisition of phosphorus and nitrogen in the rhizosphere and plant growth promotion by microorganisms. Plant Soil. 2009;321. 10.1007/s11104-009-9895-2.

[59] Badri DV, Weir TL, Van Der LD, Vivanco JM (2009). Rhizosphere chemical dialogues : plant – microbe interactions.

[60] Narula N, Kothe E, Behl RK (2009). Role of root exudates in plant-microbe interactions. J Appl Bot Food Qual.

[61] Galili G, Amir R, Fernie AR (2016). The regulation of essential amino acid synthesis and accumulation in plants. Annu Rev Plant Biol.

[62] Radhakrishnan R, Kang S-M, Baek I-Y, Lee I-J (2014). Characterization of plant growth-promoting traits of Penicillium species against the effects of high soil salinity and root disease. J Plant Interact.

[63] Jones DL, Healey JR, Willett VB, Farrar JF, Hodge A (2005). Dissolved organic nitrogen uptake by plants—an important N uptake pathway?. Soil Biol Biochem.

[64] Khan AL, Waqas M, Asaf S, Kamran M, Shahzad R, Bilal S (2017). Plant growth-promoting endophyte Sphingomonas sp. LK11 alleviates salinity stress in Solanum pimpinellifolium. Environ Exp Bot.

[65] Kang S-M, Khan AL, Waqas M, You Y-H, Hamayun M, Joo G-J, et al. Gibberellin-producing Serratia nematodiphila PEJ1011 ameliorates low temperature stress in Capsicum annuum L. Eur J Soil Biol. 2015;68.

[66] Park Y-G, Mun B-G, Kang S-M, Hussain A, Shahzad R, Seo C-W, et al. Bacillus aryabhattai SRB02 tolerates oxidative and nitrosative stress and promotes the growth of soybean by modulating the production of phytohormones. PLoS One. 2017;12. 10.1371/journal.pone.0173203 PMC5345817 28282395

[67] Egamberdieva D (2009). Alleviation of salt stress by plant growth regulators and IAA producing bacteria in wheat. Acta Physiol Plant.

[68] Kang S-M, Waqas M, Shahzad R, You Y-H, Asaf S, Khan MA, et al. Isolation and characterization of a novel silicate-solubilizing bacterial strain Burkholderia eburnea CS4–2 that promotes growth of japonica rice (*Oryza sativa* L. cv. Dongjin). Soil Sci Plant Nutr. 2017;63.

[69] Amies CR (1967). A modified formula for the preparation of Stuart’s transport medium. Can J Public Heal Can Sante’e Publique.

[70] Wang Z, Solanki MK, Pang F, Singh RK, Yang L-T, Li Y-R (2017). Identification and efficiency of a nitrogen-fixing endophytic Actinobacterial strain from sugarcane. Sugar Tech.

[71] Shahzad R, Waqas M, Khan AL, Asaf S, Khan MA, Kang S-M, et al. Seed-borne endophytic Bacillus amyloliquefaciens RWL-1 produces gibberellins and regulates endogenous phytohormones of Oryza sativa. Plant Physiol Biochem. 2016;106. 10.1016/j.plaphy.2016.05.006 27182958

[72] Chen S, Gollop N, Heuer B (2009). Proteomic analysis of salt-stressed tomato (Solanum lycopersicum) seedlings: effect of genotype and exogenous application of glycinebetaine. J Exp Bot.

[73] Manai J, Gouia H, Corpas FJ (2014). Redox and nitric oxide homeostasis are affected in tomato (*Solanum lycopersicum*) roots under salinity-induced oxidative stress. - J. Plant Physiol.

[74] Kim A-Y, Shahzad R, Kang S-M, Seo C-W, Park Y-G, Park H-J, et al. IAA-producing Klebsiella variicola AY13 reprograms soybean growth during flooding stress. J Crop Sci Biotechnol. 2017;20.

[75] Khan AL, Al-Harrasi A, Shahzad R, Imran QM, Yun B-W, Kim Y-H (2018). Regulation of endogenous phytohormones and essential metabolites in frankincense-producing Boswellia sacra under wounding stress. Acta Physiol Plant.

[76] Bilal S, Khan AL, Waqas M, Shahzad R, Kim I-D, Lee I-J (2016). Biochemical constituents and in vitro antioxidant and anticholinesterase potential of seeds from native Korean persimmon genotypes. Molecules..

[77] Shahzad R, Khan AL, Bilal S, Asaf S, Lee I-J. Plant growth-promoting endophytic bacteria versus pathogenic infections: An example of *Bacillus amyloliquefaciens* RWL-1 and Fusarium oxysporum f. sp. lycopersici in tomato. PeerJ. 2017;2017. 10.7717/peerj.3107 PMC5357341 28321368

[78] Qi QG, Rose PA, Abrams GD, Taylor DC, Abrams SR, Cutler AJ. Abscisic acid metabolism, 3-ketoacyl-coenzyme a synthase gene expression and very-long-chain monounsaturated fatty acid biosynthesis in Brassica napus embryos. Plant Physio. 1998;117. 10.1104/pp.117.3.979. 10.1104/pp.117.3.979 PMC34952 9662540

