# Supplementary material for: Indole-3-acetic-acid and ACC deaminase producing Leclercia adecarboxylata MO1 improves Solanum lycopersicum L. growth and salinity stress tolerance by endogenous secondary metabolites regulation
Source: BMC Microbiol. 2019 Apr 25;19:80. doi: 10.1186/s12866-019-1450-6 (PMC6485084; doi:10.1186/s12866-019-1450-6)
Supplement: Supplementary file 1 — Table S1. GC/MS – SIM conditions used for analysis and quantification of the indole-3-acetic acid. (DOCX 12 kb) [file 12866_2019_1450_MOESM1_ESM.docx]

**Supplementary Table.** GC/MS – SIM conditions used for analysis and quantification of the indole-3-acetic acid

| Equipment | Hewlett-Packard 6890, 5973N Mass Selective Detector |
| --- | --- |
| Column | HP-1 capillary column (30m×0.25mm i.d. 0.25µm film thickness) (J & W Scientific Co., Folsom, CA, USA) |
| Carrier gas | He (60 ml/min.); head pressure of 30 kPa |
| Source temp. | 230°C |
| Oven conditions | 70°C (2min.) → 20°C/min. → 280°C (5min) |
| Injector temp. | 200°C |
| Ionizing voltage | 70 ev |
