# Supplementary material for: Indole-3-acetic-acid and ACC deaminase producing Leclercia adecarboxylata MO1 improves Solanum lycopersicum L. growth and salinity stress tolerance by endogenous secondary metabolites regulation
Source: BMC Microbiol. 2019 Apr 25;19:80. doi: 10.1186/s12866-019-1450-6 (PMC6485084; doi:10.1186/s12866-019-1450-6)
Supplement: Supplementary file 2 — Table S2. Two-way ANOVA table of the biochemical analysis performed for tomato plants with and without MO1 inoculation and GB treatment under normal and 120 mM NaCl stress. (DOCX 18 kb) [file 12866_2019_1450_MOESM2_ESM.docx]

**Supplementary Table 2.** Two-way ANOVA table of the biochemical analysis performed for tomato plants with and without MO1 inoculation and GB treatment under normal and 120mM NaCl stress.

| **Analysis** | **Treatment** | | | **120mM NaCl** | | | **INTERACTION** | | |
| --- | --- | --- | --- | --- | --- | --- | --- | --- | --- |
|  | MS P value Variance % | | | MS P value Variance % | | | MS P value Variance % | | |
| ABA | 327700 | <0.0001 | 54.53 | 506600 | <0.0001 | 42.81 | 5408 | 0.0801 | 0.91 |
| Glucose | 20.97 | <0.0001 | 42.08 | 53.35 | <0.0001 | 53.54 | 0.9231 | 0.0369 | 1.85 |
| Sucrose | 49.52 | <0.0001 | 31.86 | 202.6 | <0.0001 | 65.18 | 1.597 | 0.0779 | 1.03 |
| Fructose | 43.61 | <0.0001 | 50.36 | 83.57 | <0.0001 | 48.25 | 0.0856 | 0.6435 | 0.10 |
| Chlorophyll contents | 25400 | <0.0001 | 77.36 | 898.9 | 0.0597 | 1.37 | 5737 | <0.0001 | 17.47 |
| Serine | 0.5713 | <0.0001 | 17.76 | 4.889 | <0.0001 | 76.01 | 0.148 | 0.0003 | 4.63 |
| Glycine | 0.0020 | <0.0001 | 85.54 | 0.00048 | <0.0001 | 10.25 | 0.000015 | 0.3584 | 0.66 |
| Methionine | 0.00022 | <0.0001 | 57.11 | 0.00018 | 0.0007 | 17.50 | 0.000078 | 0.0041 | 15.22 |
| Threonine | 0.5846 | <0.0001 | 58.61 | 0.4470 | <0.0001 | 22.41 | 0.01613 | <0.0001 | 16.17 |
| Proline | 4.978 | <0.0001 | 7.10 | 125.6 | <0.0001 | 89.56 | 1.938 | <0.0001 | 2.76 |
| Citric acid | 0.119 | <0.0001 | 57.91 | 0.1237 | 0.0001 | 29.87 | 0.00090 | 0.8029 | 0.44 |
| Malic acid | 131.8 | <0.0001 | 23.81 | 833.9 | <0.0001 | 75.30 | 2.852 | 0.005 | 0.52 |

Two-way ANOVA analysis *p*< 0.05 were performed for tomato plants with and without MO1 inoculation and GB treatment under normal and 120mM NaCl stress their interaction through GraphPad Prism software (version 6.01, San Diego, CA, USA).
